# Implementing for results: Program analysis of the HIV/STI interventions for sex workers in Benin

**DOI:** 10.1080/09540121.2013.784392

**Published:** 2013-06-09

**Authors:** Iris Semini, Georges Batona, Christian Lafrance, Léon Kessou, Eugène Gbedji, Hubert Anani, Michel Alary

**Affiliations:** a The World Bank, Washington, DC, USA; b Centre de Recherche du CHU de Québec, URESP, Québec, Canada; c Dispensaire IST, Cotonou, Bénin; d Faculté des Sciences Infirmières, Université Laval, Québec, Canada; e Independent Consultant, Cotonou, Benin; f Dispensaire IST, Cotonou, Benin; g Département de Médecine Sociale et Préventive, Université Laval, Québec, Canada; h Institut National de Santé Publique du Québec, Québec, Canada

**Keywords:** HIV, sex workers, program efficiency, service delivery

## Abstract

HIV response has entered a new era shaped by evidence that the combination of interventions impacts the trajectory of the epidemic. Even proven interventions, however, can be ineffective if not to scale, appropriately implemented, and with the right combination. Benin is among the pioneering countries that prioritized HIV prevention for sex workers and clients early on. Effective implementation up to 2006 resulted in consistent condom use among sex workers increasing from 39% to 86.2% and a decline in prevalence of gonorrhea from 5.4% to 1.6%. This study responds to the growing concern that, although proven interventions for female sex workers (FSWs) were expanded in Benin since 2008, indicators of coverage and behaviors are far from satisfactory. The quest to better understand implementation and how to render service delivery efficient and effective resonates with increased emphasis in the international arena on return for investments. Quantitative and qualitative methods were utilized to collect data. The output measured is the number of sex workers seeking Sexually Transmitted Infection (STI) care at user-friendly STI Clinics (SCs). Data were collected for 2010–2011 in nine regions of Benin. While recognizing that commitment to scale up is commendable, the study revealed deficiencies in program design and implementation that undermine outcomes. The selected mix of interventions is not optimal. Allocation of funds is not proportionate to the needs of FSW across regions. Only 5 of 41 SCs were fully functional at time of study. Free distribution of condoms covers only 10% of needs of FSWs. Funding and financing gaps resulted in extended interruptions of services. Successful HIV prevention in Benin will depend on the effective and efficient implementation of well-funded programs in sex work setting. Resources should be aligned to local sex work typology and presence in communities. A national framework defining an appropriate mix of interventions, management structure, referral mechanisms, and operational standards is required to guide rigorous implementation. Health services, in particular functional and user-friendly SCs coupled with mechanisms that link community-based work and health facilities should be strengthened to ensure STI care/anti-retroviral treatment expansion. Without leadership of sex workers, any attempt to end HIV will be unsuccessful.

## Introduction

The response to the human immunodeficiency virus (HIV) epidemic has entered a new era, significantly shaped by emerging evidence on the effect that combinations of HIV prevention interventions have on the trajectory of the epidemic. In terms of HIV transmission via sex work, a combination of interventions that promote education, condom use, sexual health, solidarity, empowerment, and rights for sex workers is effective and has averted three million HIV infections in India (Kumar et al. 2011) and contained HIV epidemics in Benin (Boily, Lowndes, & Alary, 2002), Thailand, and Cambodia.

Nevertheless, experience has demonstrated that successful replications of multidimensional prevention interventions with integrated links to treatment across the country depend on program delivery (Padian et al., 2011). Thus, interventions that have proven to be effective in preventing HIV/Sexually Transmitted Infection (STI) transmission, can be ineffective if implemented incorrectly, or not at the required scale, or with the right combination.

In this context, this study investigates the program design and the implementation efficiency of the HIV/ STI programs in the sex work context in Benin. Benin is a low-income country with a population estimated at 9.6 million (2011), life expectancy at birth is 56 years old, and 33.3% of the population lives below the poverty line. Despite growth rates averaging about 4% annually during the last decade, poverty remains widespread and youth unemployment is high. The 2011–2015 growth- and poverty-reduction strategy focuses on education and health (The World Bank, 2011).

Since the HIV epidemic emerged in Benin, men and women involved in sex work were the most affected by the epidemic accounting for the majority of HIV infections in the country. The Canadian International Development Agency (CIDA) funded and jointly with the Government of Benin implemented HIV prevention projects for female sex worker (FSW) and clients, which culminated with SIDA-3 project in 2001–2006. These efforts were effective: from 1998 to 2005, consistent condom use by FSWs increased from 39.0% to 86.2%, and the prevalence of gonorrhea decreased from 5.4% to 1.6% (Lowndes, Alary, & Labbe, et al., 2007). The HIV prevalence among FSWs declined from a peak of 55% in 1999 to 24.7% in 2011 (The World Bank, CNLS-Benin, 2011).

In 2006, as its SIDA-3 funding ended, the Government of Benin maintained momentum with resources from the World Bank, the US Agency for International Development, and the Global Fund for AIDS, tuberculosis, and malaria. The SIDA-3 service delivery models for FSWs were expanded at the national level, however, indicators of coverage and behavior indicate lags in implementation: HIV prevalence in the general population (1.2% in 2006) and among pregnant women has stabilized over the past 10 years, HIV prevalence among FSW continues to be at least 20 times higher than HIV prevalence in the general population, and 3.7% of FSW clients are estimated to be HIV infected (The World Bank, CNLS-Benin, 2011).

In 2009, only 56% of FSW were reached with HIV prevention programs (CNLS-Benin, 2010) and only 0.6% of HIV-related resources were spent on HIV programs for sex workers (UNAIDS, the World Bank, 2010). In a 2008 study, approximately 79.7% of FSW used condoms with their paying clients on the last day they worked, compared to 82.8% in 2005. The decline has been attributed to the loss of SIDA-3 (The World Bank, 2012).

Against this background, this study aims to analyze implementation of the HIV/STIs programs in sex work setting. Recommendations are presented to inform future program design, improve efficiency of resource allocation and how to render efficient and effective implementation delivery scaled up at national coverage.

## Study design

**Figure 1.**
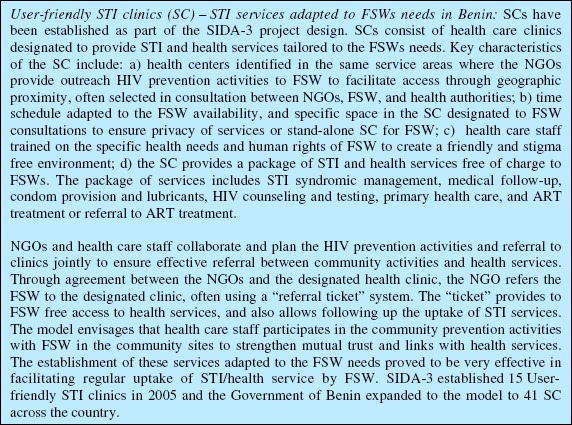
Description of the user-friendly STI clinics.

The study was designed to analyze implementation efficiency through (1) normative analysis of HIV program design in sex work context; (2) service delivery efficiency; and (3) unit cost per output. Evaluation through normative analysis compares what is taking place to what should be taking place, it assesses activities and achievement of targets (Gertler, Martinez, Premand, Rawlings, & Vermeersch, 2011). For the purpose of this study, normative analysis is applied to the program design, operational characteristics of implementation, and outputs, through benchmark comparison with SIDA-3 experience in Benin (2006) and the Avahan, the Bill and Melinda Gates Foundation's initiative to reduce the spread of HIV in India (Avahan and Bill & Melinda Gates Foundation, 2008). Indicators selected to measure implementation frequency include (1) number of outreach workers (OW) and peer educators (PE) per population of FSWs served in their catchment area, (2) number of awareness sessions with FSWs per month, and (3) number of condoms distributed. User-friendly STI Clinics (SCs) functionality was measured through for January 2010–June 2011: (1) nonfunctional, if there were no record of FSW attendance; (2) limited functionality, if there were less than 10 FSW visits; and (3) functional, if there were records of 10 or more FSW visits (Figure 1).

Efficiency consists of delivering the maximum output with the least cost, measured through the output and unit costs (Weir et al, 2012). The output in this study is the *number of sex workers seeking STI care in SCs and designated health clinics.* Community-based unit costs of FSW visit to STIs services were measured through the top-down approach of budget allocated to nongovernmental organizations (NGOs) per number of FSW visits. Bottom-up approach was used to measure unit costs per FSW visit in SC in Cotonou and Bohicon. Quantitative methods and observations were utilized to collect the primary data. Qualitative methods were applied to obtain the perspective of SW and service providers.

### Sample

A nonprobabilistic purposive sampling was adopted. Nine of the 12 regions of Benin were selected for data collection of activities that occurred from January 2010 to September 2011, including regions where SIDA-3 had operated and regions where it had not. The cities of Cotonou, Porto-Novo, Parakou, and Bohicon were included as these cities host the majority of Benin's sex workers. From 41 existing SCs, 23 were selected in the nine regions to best represent the variety of activity levels and diverse management practices, including SCs integrated into health facilities and SCs integrated into NGO or other organization. Thirty-three community-based organizations (CBOs) were selected out of 55 NGOs implementing community-based interventions for FSW.

### Data collection

Data were collected at the CBOs, SCs, and antiretroviral treatment (ART) centers using pre-designed, field-tested quantitative tools. Quantitative data for program description and normative analysis were collected through mapping questionnaires and on-site observations. Secondary data included on-site programmatic, service records and financial reports at delivery points as well as at regional and headquarters levels to extract the data on service utilization. Qualitative interviews with FSWs, service providers, and managers of HIV projects were conducted to provide insights into program implementation.

### Data analysis

Quantitative data were stored and analyzed in MS Excel to generate information on service frequency, intensity, and utilization indicators. The mean costs per FSW visit were compared between clinics with high client volume and those with low client volume using t-test with the SAS statistical package (SAS Institute Inc., Cary, NC, USA). Qualitative analysis was undertaken using Bardin (Bardin, 1986) and L'Ecuyer (L'Ecuyer, 1988) methods. Grouping by category of strength and weaknesses per site allowed identifying operational characteristics. Data interpretation was done through triangulation for cross-comparison of empirical data, the observations, and the descriptive and comparative reviews. Triangulation served to verify the validity of the findings, key features identified as strength and weaknesses that influence efficient service delivery. It reduced bias generated through individual methods of data collection.

## Results

Community-based services and SCs have been established and funded in all 12 regions of Benin. The HIV prevention programs for FSW are funded mainly through external resources. Each donor defines their own package of services and implementation standards. Our study revealed the absence of a standardized package of HIV/STI interventions for FSWs, clients, and partners in Benin.

**Figure 2.**
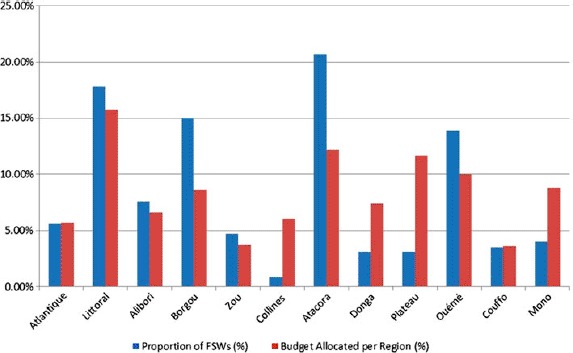
Budget allocation compared to proportion of FSWs per region.

Allocated funds per region are disproportionate to the presence and the needs of FSW. Borgou and Atacora, which host a large population of FSW, have limited resources compared to Colline, Donga, Plateau, and Mono, which have more limited numbers of FSW (Figure 2).

*The programs* provide services to overt and clandestine FSWs. There are no interventions targeting emerging categories of sex workers (those working through phone contacts), the clients, and sexual partners. Male sex workers are not included, although their presence has been documented. The interventions implemented include community-based prevention, referral to designated SCs and health clinics, STI and voluntary counseling and testing (VCT) provision, and other health services. Interventions that tackle structural issues, individual stigma, sex work community mobilization, or create an enabling environment to alter the social context of risk and violence are almost nonexistent. Most of the contractual agreements with the NGOs run for either three to six months or 12 months. Financing irregularities have resulted in three- to six-month interruptions of community-based services for FSW across the country.

Behavior change communication (BCC); information, education, and communication activities; condoms and lubricant distribution at community-level sites; referral to the designated SCs, VCT, and health services are included in all contractual agreements with 55 NGOs. Community-based outreach is implemented through FSW PEs. Each NGO has two OWs and 5–20 PEs regardless of the number of FSW to be reached. Delays in the provision of incentives (1000–2000 CFA per awareness session) coupled with high mobility of FSWs has resulted in a decrease in the number of the FSW PEs: 234 had been trained at the start of the review period but 181 were actually working. On average, each PE conducts or four to eight sessions per month, while OW conduct bimonthly supervision and training of PEs, significantly less than 20 sessions per month implemented by PEs in the SIDA-3 project.

The number of male condoms distributed falls short of the needs of FSW. The NGOs should hypothetically provide three condoms per contact; the records indicate that the NGOs distributed approximately 1.5 condoms per contact. Free distribution of condoms covers only 10% of FSW needs, while there is lack of lubricants.

Only 5 out of 41 SCs were fully functional at the time of the study. Seventeen of the SCs had limited operational functioning, and 19 were nonfunctional. Two of the five functional centers are NGO owned and run, and three are hosted in the public health centers. In almost all 23 centers visited for this study, staff was trained to provide user-friendly services to FSWs. Considerable recurrent shortages of STI drugs and equipment, such as speculums, were observed in all the adapted STI services, affecting service availability and quality – managed and supplied by the central level. VCT is integrated in all SC. Use of HIV rapid tests has increased the rate of obtaining responses to 95–98%. Where the service is not offered in-house, the FSWs are referred to the closest VCT center. There is no system to provide feedback on those who take the test. Ad hoc refresher training courses for staff and irregular management supervision were seldom undertaken from the central and district levels.

**Figure 3.**
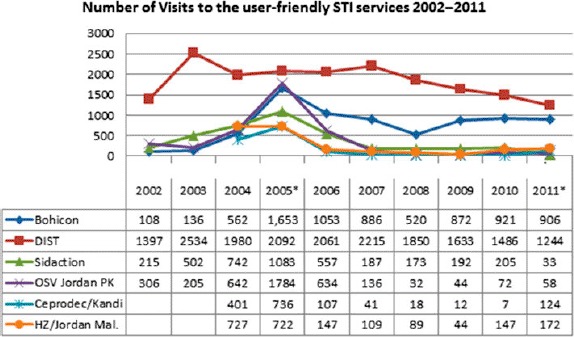
Number of visits of FSWs to the user-friendly STI services 2002–2011. Note: *Data for January–September 2011.

The number of contacts with FSW is less than 50% of that reached by SIDA-3 in 2005. With the exception of the Cotonou and Bohicon centers, the STI and other health services utilization by FSW have been in decline compared to SIDA-3 indicators (Figure 3). Only 47% of those referred from NGOs seek health services in the clinics, denoting a significant disconnect between community-based work and uptake of SCs services. In 2010, 1186 new FSW registered and 3372 visits were recorded in all SCs. The 3372 FSW visits to 41 centers in 2010 denote a decrease of more than 50% as compared to 8809 visits to 14 SCs in SIDA-3 2005. Ninety percent of the visits were registered in SCs in six of the main cities, which host the majority of FSWs. Monthly visits increased slightly in 2011 over 2010, with 326 average visits and 281 visits, respectively (Table 1).

**Table 1.** Service availability and utilization by FSW in 2010–2011.

							Type of STI center		Type of services available			Number of visits of FSW	
Region	Administrative unit	Number of FSWs (2008)	STI center	NGO	Government	STI	VCT	ARV	2010	2011^a^
Alibori	Kandi	341	CEPRODEC Kandi	x		x	x		7	124
			Kandi/		x	x			57	33
	Banikoara	140	Banikoara		x	x			29	0
	Gogounou	148	–			x
	Malanville	363	OSV Jordan (Malanville)	x		x	x		147	172
Atacora	Kérou		CS Kérou		x	x	x	x	0	0
	Natitingou	1327	CS Natitingou		x	x			12	25
	Péhunco	38	CS Péhunco		x	x	x	x	0	0
Atlantique	Allada	18	HZ Allada		x	x	x	x	0	0
	Ouidah	102	Ouidah		x	x	x	x	0	0
	Abomey-Calavi	605	–
Borgou	Kalalé	446	Kalalé		xx		0	0
	N'dali	14	N'dali		x	x	x	x	1	0
	Tchaourou	248	Tchaourou		x	x	x		0	0
	Parakou	1023	Zongo 2/Govt.		x	x			109	74
			OSV Jordan	x		x	x	x	72	58
Collines	Glazoué	27	Glazoué		x	x	x	x	44	91
	Paouignan		Paouignan		x	x	x		36	13
	Savè	2	Savè		xx		5	0
	Tchetti	–	Tchetti		x	x	x		0	0
Couffo			APH Gohomey			x	x	x	0	0
	Aplahoué	284	Aplahoué		x	x			22	0
	Dogbo	29	MSF Dogbo (SONAGNON)	x		x	x	x	0	0
	Klouékamè	56	Klouékamè		x	x	x	x	0	–
Donga	Djougou	248	Djougou		x	x	x	x	7	8
	Kolokondé		Kolokondé		x	x			13	4
Littoral	Cotonou	2423	Cotonou 1(STI Center)		x	x	x	x	1486	1634
			Gbegamey		x	x			0	0
			Houénoussou		x	x			0	0
			Placodji		x	x			0	0
			St Pothin (Cotonou)	x					0	0
Mono	Agamey	–	Agamey		x	x			0	0
	Bopa	17	Bopa		x	x	x	x	0	0
	Comé	90	Comé		xx		0	0
	Lokossa	275	Lokossa-HZ Lokossa		x	x	x	x	12	5
Ouémé	Avrankou	218	CS Avrankou		x	x	x	x	79	29
	Porto-Novo	980	Oganla		**X**	**X**	**X**	**X**	55	46
			ONG SIDACTION		**X**	**X**	**X**		205	33
Plateau			Igana		**X**	**X**			42	55
	Kétou	166	Kétou		**X**	**X**	**X**	**X**	11	12
Zou	Abomey	104	Abomey		**X**	**X**	**X**		0	0
	Bohicon	475	Bohicon		**X**	**X**	**X**		921	1269
	Covè	5	Covè		**X**	**X**	**X**	**X**	0	0
Total									3372	4707

Note: ^a^Data for January–September 2011.

ART services are mainstreamed in 18 SCs. Data in three centers indicate that 15–33% of those on treatment are FSWs. The other centers had no record of FSW seeking neither STIs nor VCT or ART services (Table 2).

**Table 2.** Summary of data on HIV testing and the number of HIV positive FSWs on ART by the end of September 2011.

	ART centers		
Indicators^a^	STI Center Cotonou	Sèmè Kpodji	Grand-Popo
Number of FSWs HIV tested	271^a^	–	41
Number of FSWs HIV positive	26^a^	–	–
Percentage of FSWs on ART treatment out of those tested	34	101 (absolute nb)	2
Number of HIV-positive women on ART treatment	283	170	17
Percentage of FSWs among women on ART	32	59	10
Number of HIV-positive men on ART	114	103	05
Total number of people on ART	397	273	22

Note: ^a^Data for January–September 2011.

### Unit costs

The NGO cost per FSW visit seeking STI services in clinic varied, with an overall figure of US$61 per FSW visit. Where is high volume of FSWs visits, the overall NGO cost per FSW visit to the clinic was US$28.5, with a variation from US$7 to US$103. This was significantly lower in low-volume clinics (*p* = 0.039), where the overall NGO cost per FSW visit was US$481.7. The average total (NGO, clinical, surveillance and management) cost per FSW visit to the SC in Bohicon was US$28.6, while the same cost in the Cotonou Center was US $46.9, similar to the unit costs of the Avahan project.

## Discussion

It is commendable that the government and partners have expanded HIV services for FSW at national coverage. However, commitment and service availability, even at national scale, do not necessarily translate into health outcomes. Our study revealed deficiencies in program design and implementation that have led to decreased number of FSWs reached with HIV prevention and treatment services over the years.

The mix of interventions implemented is not satisfactory. A combination of behavioral, structural, and biomedical approaches, implemented with the wisdom and ownership of communities, offers the best hope for successful prevention (Herson et al., 2008). The current programs target the individual risks of FSW. However, the SIDA-3 and Avahan experience suggest that tackling exclusively the individual risk is likely to have a limited impact. Structural interventions that address societal causes like social exclusion, police harassment, stigma, and legal environment coupled with program ownership by FSWs and communities, may remove barriers for access to services and enable FSW to have greater control of their work condition and seek health services. Structural interventions that sought to reduce individual vulnerability, create an empowering and enabling environment, and facilitate access by FSW to STI/HIV services have been effective in India, Brazil, and elsewhere and need to be part of the programmatic package (Biradavolu et al., 2012; Cohen, 2004; Kerrigan et al., 2012). Adapted strategies to reach clients and sexual partners of FSWs, which yielded positive results during the SIDA-3 implementation in Benin (Lowndes et al., 2007), also need to be integrated.

Planning that does not take into account local evidence might result into reaching with services only selected sub-populations of FSW. Anecdotal evidence indicates that there is a diversification of sex work typology, including FSW operating through mobile phones and from other settings, while current response focuses only overt and clandestine FSWs (PNLS-Benin, 2009). This is compounded by the mismatch of allocated resources and FSWs needs at the regional level. Operational research is required to map and better understand the emerging trends of sex work and local dynamics (Wilson, 2005), and translate the findings into locally adapted strategies with commensurate funding allocations per region.

**Figure 4.**
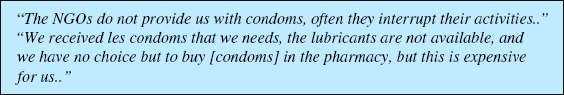
Quotes from sex workers.

The decrease in the uptake of STIs services can be attributed to considerable dysfunctional SCs coupled with insufficient frequency and intensity of outreach interventions. The number of PEs and OWs and the number of the BCC sessions with FSW is set by the donor rules and not by the targeted population size, which has led to insufficient time and personal to conduct outreach activities. The Avahan experience indicates a more successful ratio of at least one PE per 50 FSW (Avahan, 2010). The number of BCC sessions by PEs with FSWs is significantly less than 20 sessions per month implemented by PEs in the SIDA-3 project, an agreed upon standard for outreach. The Avahan experience has demonstrated that program implementation can be effective only if each partner has a core set of program interventions to implement, which determines the combination of interventions, management, and incentives. The variations in the number of PEs, OWs, and sessions per month can be addressed through a national operational standard framework that defines the frequency and intensity indicators of community-based activities, sets quality standards for all partners, and saturate the community with locally tailored services, condoms, and lubricants. Condom and lubricant provision to saturate the needs of the FSW shall be planned and included in the package of services (Figure 4).

**Figure 5.**
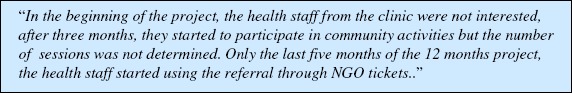
Quote from NGO staff.

It is essential that SCs are strengthened to ensure quality provision of STI and health services to FSWs. Another negative effect of low uptake of STI services is the limited uptake of ART by FSW. Although ART services are mainstreamed in 18 SCs, the disconnect between community activities and health services results in limited utilization of STIs, VCT, and subsequently of the ART (Figure 5). While previous projects in Benin mainly focused on linking community activities with STIs services, given the recent biomedical advances related to treatment-as-prevention (Cohen et al., 2011), it is also critical to improve linkages to ART adherence and ongoing prevention for FSW living with HIV.

The low uptake of STIs and ARTs services warrants particular attention, as it is an indication of inefficiency of service delivery. Studies suggest that volume predicts 25–95% of variations in efficiency (Marseille, Dandona, & Marshall et al., 2007), while Kumaranayake indicates that volume variations explains between 50% and 70% of the variations in costs across facilities for the same intervention (Kumaranayake, 2008).

Similarly, high volume of STI and ART services’ utilization in Cotonou and Bohicon centers can be one of the main factors for unit costs significantly lower than other centers that have served only a few FSWs. In both centers, these results were attributed to regular interactions between health service providers, community-based actors, and FSWs. Mechanisms that optimize the synergy between community-based work and health facilities are essential component to ensure STIs and ART expansion among FSWs through community outreach and social mobilization strategies.

Limited capacity to supervise, manage, and coordinate is another added challenge. Effective delivery of HIV prevention interventions is the result of integrated community-based actions and health services, which are not part of the same management system. The programmatic expansion in Benin was not accompanied with either additional personal to manage national-level HIV prevention programs or with capacity strengthening in management skills. Making the system work will necessitate a management structure (Berthozzi et al, 2008) and dedicated human resources from national level to grass-root organizations with appropriate skills to effectively translate managerial decisions into actions at point of delivery. Effective implementation will require regular technical support coupled with efficiency and effectiveness analysis to systematically improve performance of service delivery. Regular funding to community actors through contractual agreements of at least 12 months should be adopted.

One of our study limitations is that the method and practices of data registration may actually underestimate the utilization of STI services by FSWs. Current monitoring tools do not capture coverage; they record the number of contacts with FSW but they do not systematically record the number of FSWs reached. The monitoring system does not capture the FSW as unique individual, therefore, our study could not measure the overlap of data caused by FSW mobility between sited. The current paucity of information on performance, costs, and coverage, creates inertia in implementation. Without data, program managers fail to proactively undertake adjustments tailored to the needs of FSWs.

## Conclusion

Successful HIV prevention in Benin will largely depend on effective and efficient implementation of HIV programs for sex workers, clients, and their sexual partners. This will require resource allocation aligned with the sex work needs in each region. A national framework defining an appropriate mix of interventions, referrals, management structure, and operational standards is required to guide rigorous implementation. Health services, in particular SCs, and mechanisms that link community-based work and health facilities should be strengthened to ensure STI care/ART expansion. Measuring and ensuring effective and efficient implementation need to be integrated in the response. Without leadership of sex workers, any attempt to end HIV will be unsuccessful.
